# Dexamethasone application for *in vitro* fertilisation in non-classic 17-hydroxylase/17,20-lyase-deficient women

**DOI:** 10.3389/fendo.2022.971993

**Published:** 2022-10-28

**Authors:** Xiu-Li Yang, Ting-Ting Zhang, Jing Shang, Qing Xue, Yan-Rong Kuai, Sheng Wang, Yang Xu

**Affiliations:** ^1^ Department of Obstetrics and Gynecology, Peking University First Hospital, Beijing, China; ^2^ Department of Endocrinology, Peking University First Hospital, Beijing, China

**Keywords:** 17-hydroxylase/17,20-lyase deficiency, dexamethasone, infertility, *in vitro* fertilization, pregnancy

## Abstract

**Context:**

High progesterone levels in the follicular stage interfere with the implantation window, causing infertility in women with 17-hydroxylase/17,20-lyase deficiency (17OHD). Dexamethasone can restore cortisol deficiency and suppress inappropriate mineralocorticoid secretion to control hypertension in 17OHD patients, but poses risks to the foetus if administered during pregnancy.

**Objective:**

We prospectively explored a rational glucocorticoid use protocol for assistive reproduction in a woman with non-classic 17OHD that reduced glucocorticoid side effects.

**Method:**

In this study, the treatment protocol for this 17OHD patient included the following steps. First, the appropriate type and dose of glucocorticoid for endogenous progesterone suppression was determined. Then, glucocorticoid was discontinued to increase endogenous progesterone levels for ovarian stimulation. Next, dexamethasone plus GnRHa were used to reduce progesterone levels in frozen embryos for transfer. Once pregnancy was confirmed, dexamethasone was discontinued until delivery.

**Results:**

Dexamethasone, but not hydrocortisone, reduced progesterone levels in the 17OHD woman. After endogenous progesterone-primed ovarian stimulation, 11 oocytes were retrieved. Seven oocytes were 2PN fertilised and four day-3 and two day-5 embryos were cryopreserved. After administering dexamethasone plus gonadotropin-releasing hormone agonist (GnRHa) to reduce progesterone levels to normal, hormone replacement therapy was administered until the endometrial width reached 9 mm. Exogenous progesterone (60 mg/day) was used for endometrial preparation. Two thawed embryos were transferred on day 4. Dexamethasone was continued until pregnancy confirmation on the 13^th^ day post-transfer. Two healthy boys, weighing 2100 and 2000 g, were delivered at 36 weeks’ gestation.

**Conclusion:**

Rational use of dexamethasone synchronised embryonic development with the endometrial implantation window, while not using in post-implantation avoided its side effects and promoted healthy live births in women non-classic 17OHD undergoing *in vitro* fertilisation.

## Introduction

**Figure 1:**
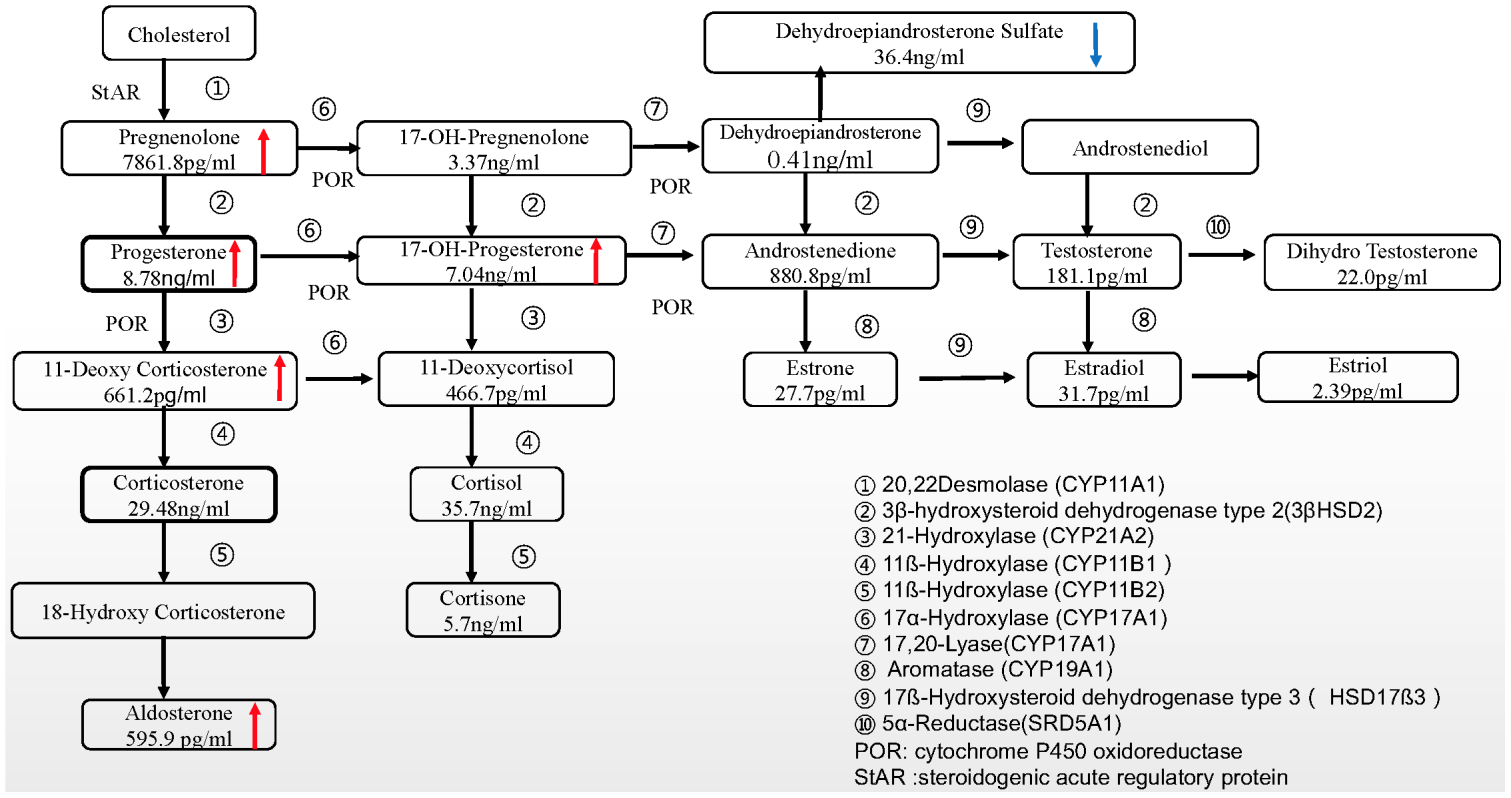
Profiles of steroid hormones detected by mass spectrometry.

17α-hydroxylase/17,20-lyase (P450c17) is a cytochrome P450 enzyme that is expressed mainly in the adrenal cortex and gonads and plays a role in both 17α-hydroxylase and 17,20-lyase activity in steroid hormone synthesis (**Figure 1**). The P450c17 is encoded by *CYP17A1* (NM-000102), located on 10q24.3. The *CYP17A1* mutations can cause 17OHD and differences in *CYP17A1* mutations determine clinical manifestations and hormone level variations. Deficiency in 17α-hydroxylase/17,20-lyase (17OHD) was first reported by Biglieri et al. in 1966 (1). Low cortisol levels in 17OHD lead to ACTH hypersecretion and in absence of P450c17, its substrates (deoxycorticosterone, 18-deoxycorticosterone, and progesterone) accumulate, with concomitant product (cortisol, androgen, and oestrogen) reduction (2).

As such, 17OHD is clinically divided into classic and non-classic types. The classic type of 17OHD is characterised by hypertension, hypokalaemia, primary amenorrhoea, and sexual infantilism, whereas non-classic cases may only show infertility (3). Elevated progesterone levels are a key characteristic of 17OHD in both classic and non-classic types (4). High progesterone levels in the follicular stage interfere with the implantation window, resulting in infertility among women with 17OHD (5, 6). The follicular development in females with classic 17OHD is arrested because of the lack of ovarian oestrogen synthesis (7). However, non-classic 17OHD patients can still conceive, because residual enzyme activity allows synthesis of low levels of oestrogen. Therefore, assisting such patients in achieving pregnancy is a clinically important. Because of the rarity of such cases and the complexity of the condition, only a few patients with non-classic17OHD have obtained live births by *in vitro* fertilisation (IVF) and frozen transfer (8–11). In the IVF process of 17OHD infertile women, glucocorticoids were routinely used to reduce elevated blood progesterone (8–12), but long-term use of glucocorticoids during pregnancy can increase the risk of side effects of mothers and fetuses, and even cause fetal malformations (12–14).

We conducted this prospective case study to explore a rational glucocorticoid use protocol for IVF in women with 17OHD, while reducing the side effects of dexamethasone in foetuses and mothers with non-classic 17OHD-related infertility.

## Methods and results

### Case characteristics

A 28-year-old infertile woman with non-classic 17OHD presented with main complaints of irregular menstruation and primary infertility. She was diagnosed with non-classic 17OHD using steroid hormone profiling (**Figure 1**), adrenocorticotrophic hormone stimulation test, and genetic testing. The patient exhibited relatively normal secondary female phenotypes. Clinical history revealed that menarche occurred at the age of 14 years, and menstruation cycles varied between 2 and 3 months and lasted 3-4 days. Appearance of spontaneous cysts on ovaries was noted intermittently. The adrenal magnetic resonance imaging results, blood pressure, and plasma potassium levels were normal. The basic maternal progesterone levels in the follicular phase (reference range: 0.31–1.52 ng/mL) are listed in **Table 1**. The low dose dexamethasone test (0.5 mg, every 6 hours for 2 days) showed marked cortisol inhibition and normal progesterone levels. Genetic testing revealed two mutations in *CYP17A1*, including c.932_939 del TTAAATGG (chr10-104592780–104592787 mat) and c.403T >C (chr10-104595044 pat). c.403T>C(p.F135L) variant was not reported in HGMD and ClinVar databases. The population frequency of c.403T>C variants was not found in gnomAD, Exac and 1000genomes databases(PM2-supporting). Through the analysis of NGS data of the family, c.932_939delTTAAATGG variant was inherited from the mother, while c.403T>C was inherited from the father, which is consistent with the in trans variants’ transmission in the autosomal recessive pattern of inheritance for 17OHD(PM3). Multiple lines of computational evidence(SIFT, PROVEAN、Polyphen-2_HDIV and Polyphen-2_HVAR) supported a deleterious effect on the gene(PP3). According to ACMG classification, c.932_939delTTAAATGG was classified as pathogenic, while c.403T>C was classified as a variant of uncertain significance.

**Table 1:** Hormone levels of the patient.

	Units	Reference values	Baseline values	Hydrocortisone			Dexamethasone	
								Before treatment	10 mg bid for 14 days	10 mg tid for 14 days	Before treatment	0.375mg Qd for 14 days
FSH	mIU/ml	3.85–8.78	5.87					
LH	mIU/ml	2.12–10.89	7.86					
E	pg/ml	27–122	32					
P	ng/ml	0.31–1.52	5.11	2.39	4.13	4.26	5.01	0.5
T	ng/ml	0.1–0.75	0.27	0.06	0.33	0.02		<0.01
PRL	ng/ml	3.34–26.72	9.58					
Cortisol	µg/ml	4.4–19.9	15.31					
ACTH	pg/ml	7.2–63.3	43.23					
K	mmol/L	3.5–5.3			3.57	3.86		
Glu	mmol/L	3.61–6.11			6.21	5.54		
17OHP	ng/ml	0.1–0.8	6.2					

FSH, follicle-stimulating hormone; LH, luteinising hormone; E, oestrogen; P, progesterone; T, testosterone; PRL, prolactin, ACTH, adrenocorticotropic hormone; Glu, glutamate; 17OHP, 17-hydroxyprogesterone; bid, twice a day; tid, three times a day.

### Treatment protocol

Due to the asynchronous development of follicles and the endometrium, we performed IVF. The treatment protocol included the following four steps. First, we determined the appropriate type and dosage of glucocorticoids for endogenous progesterone inhibition. Second, we stopped using glucocorticoids to increase endogenous progesterone levels for progesterone-primed ovarian stimulation (PPOS). Third, after ovulation induction and egg retrieval, dexamethasone plus GnRHa were used to reduce progesterone levels for frozen embryo transfer. Fourth, we discontinued dexamethasone administration once pregnancy was confirmed, until delivery.

### Determination of the appropriate glucocorticoid type and dosage for endogenous progesterone inhibition

In this case study, use of hydrocortisone (Xinyi Pharmaceutical Co., Ltd, Shanghai, China) to reduce progesterone levels was first tested because of its mild side effects on foetuses. However, the progesterone level did not reduce successfully, even after increasing the drug dose to 30 mg/day. Hydrocortisone was discontinued when the patient developed Cushing’s symptoms (facial rounding and weight gain). Two months later, the medication was switched to dexamethasone (Lisheng Pharmaceutical Co. Ltd. Tianjin, China), starting with a low dose (0.375 mg/day at bedtime). After 14 days, her progesterone level dropped to 0.5 ng/ml, without obvious adverse reactions. We chose dexamethasone 0.375 mg daily to control the patient’s pre-transfer progesterone level. The hormone changes are listed in **Table 1**.

### Use of endogenous progesterone to block the luteinising hormone surge without glucocorticoid application

After the dose and type of glucocorticoid were determined, dexamethasone administration was stopped. This resulted in an increase in endogenous progesterone (5.07 ng/ml) in the follicular phase. Human menopausal gonadotropin (hMG; Menopur, Ferring Pharmaceuticals, Saint-Prex, Switzerland) and urofollitropin for injection (uFSH, Livzon Pharmaceutical Group, Inc., Zhuhai, China) were used for 13 days of controlled ovarian stimulation. The hormone levels achieved were listed in **Table 2**. This treatment resulted in two dominant follicles, approximately 18 mm in size. We used 250 µg of Ovitrelle (Merck, Darmstadt, Germany) to induce oocyte maturation. Thirty-six hours later 11 oocytes were retrieved. Seven oocytes underwent normal 2PN fertilisation, of which four day-3 embryos and two day-5 embryos were cryopreserved.

**Table 2:** Hormone levels during controlled ovarian hyperstimulation.

	D2	D6	D8	D11	D12	D13	D14
FSH (mIU/ml)	5.6	8.13	8.76	8.55	12.63	15.98	17.22
LH (mIU/ml)	9.11	4.58	2.76	1.92	1.47	1.33	1.34
E2 (pg/ml)	43	275	663	1114	1187	1395	2186
P (ng/ml)	5.07	3.15	4.17	3.34	3.57	4.02	5.47

D, The menstrual cycle day in controlled ovarian stimulation; FSH, follicle-stimulating hormone; LH, luteinising hormone; E2, oestradiol; P, progesterone.

### Dexamethasone plus GnRHa reduce endogenous progesterone for hormone replacement therapy

After the first menstrual cycle post-retrieval, we used long-acting GnRHa—triptorelin acetate (Dophereline, Ipsen, Paris, France) 3.75 mg once, followed by 0.375 mg dexamethasone daily to reduce endogenous progesterone, as hormone replacement therapy prior to frozen embryo transfer. One month later, the patient’s progesterone level had reduced to 0.04 ng/ml. Oral oestradiol was used until the endometrial width reached 9 mm. During this period, the blood progesterone level was 0.21 ng/ml. Therefore, exogenous progesterone (60 mg/day) was administered to prepare the endometrium. On the 4th day of progesterone supplementation, two thawed embryos were transferred.

### Discontinuation of dexamethasone from pregnancy confirmation until delivery

**Figure 2:**
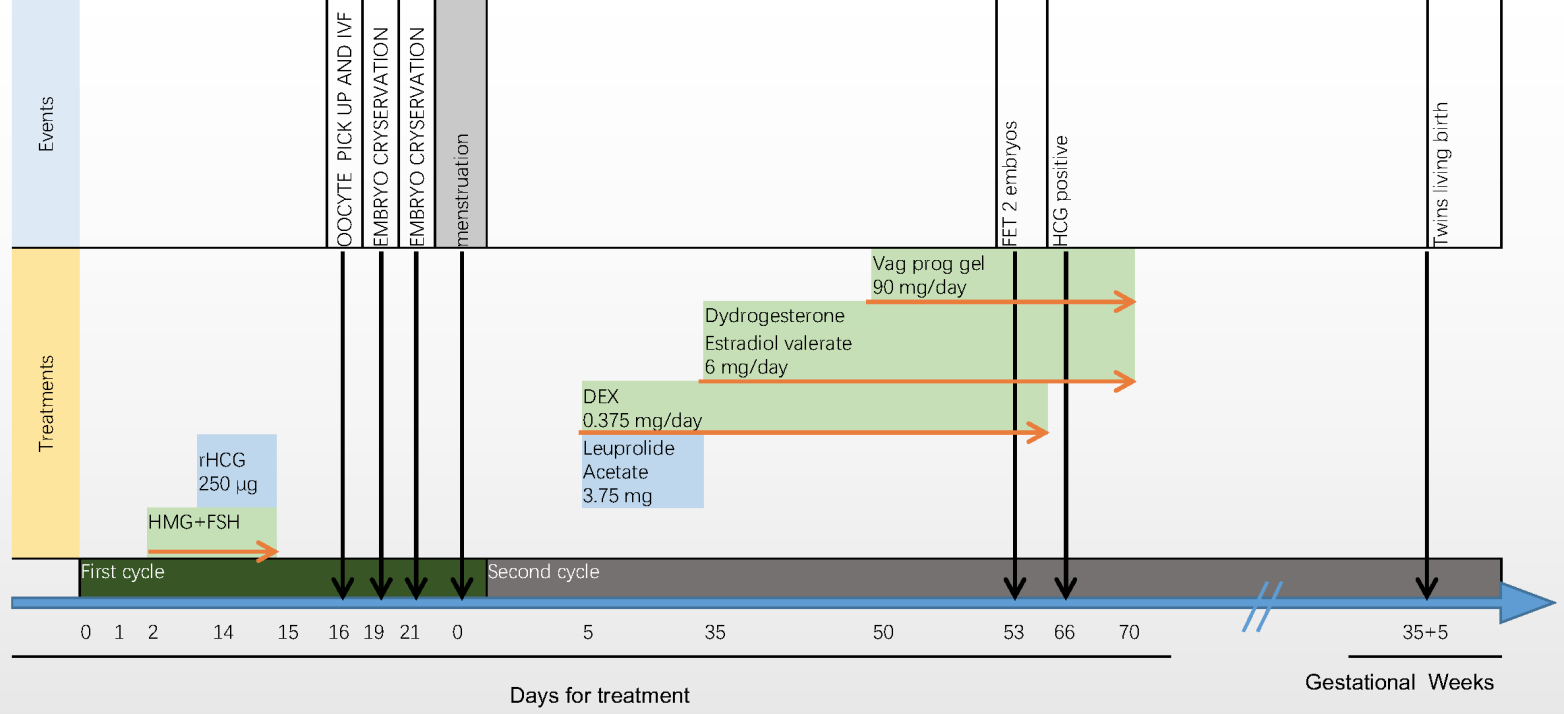
Introduction of the treatment process. The oestradiol concentration moderately increased from 43 pg/ml to 2186 pg/ml, and the progesterone concentration remained relatively constant from 3.15 ng/ml to 5.47 ng/ml. After triggering by human chorionic gonadotrophin (hCG), 11 oocytes were retrieved, of which seven were fertilised. Four day-3 and two day-5 embryos were cryopreserved. Leuprolide acetate (3.75 mg daily) and a low dose of dexamethasone (0.375 mg daily) were administered in the first cycle after ovum pick-up (OPU). One month later, 14 days after oestradiol valerate administration, the endometrial width reached 9 mm. Exogenous progesterone (60 mg daily for 3 days) was added, and frozen–thawed embryo transfer was performed. After 3 days, two embryos (8/II/1 and 7/II/1) were transferred.

Thirteen days after embryo transfer, when serum human chorionic gonadotropin (hCG) was 864.25 IU/ml, dexamethasone use was discontinued. By 28 days after IVF, transvaginal ultrasound examination detected viable pregnancy of twins in the uterus. During early pregnancy, as the fasting blood glucose level was slightly elevated, insulin was used to control the blood glucose to the target level. The patient’s blood pressure remained normal during pregnancy. The ultrasound results throughout pregnancy were normal. In the 36th week of pregnancy, two healthy baby boys were delivered, weighing 2100 g and 2000 g. We have been following the postnatal development of the twins, and their motor and language developments were normal so far (15 month old). The introduction of the treatment process are listed in **Figure 2**.

## Discussion

This report highlights the rational use of glucocorticoid, particularly dexamethasone rather than hydrocortisone, in women with 17OHD undergoing IVF. First, this process includes the use of endogenous high progesterone levels to promote ovulation without the use of glucocorticoids. Second, we used dexamethasone to reduce endogenous progesterone levels for hormone replacement therapy before transfer of frozen embryos. Glucocorticoid use was discontinued from pregnancy confirmation to delivery. This approach facilitated the birth of healthy twin boys to an infertile woman with non-classic 17OHD.

High progesterone levels disturb the embryo implantation window. Thus, progesterone levels need to be reduced during embryo transfer (5). However, high progesterone levels are not a barrier to controlled ovarian hyperstimulation. Previous studies have shown that ovarian stimulation could be performed under the blockade of endogenous or exogenous progesterone to suppress early onset LH peaks (15). Progesterone levels higher than 2 ng/ml before oestrone administration are sufficient to inhibit the positive action of oestrogen on LH release (16–18). In this study, we stopped applying glucocorticoid pre-stimulation to allow return of progesterone to high levels to perform the PPOS protocol. The protocol presented in this study resulted in relatively high oestrogen levels and qualified embryos. We propose that this is an appropriate controlled ovarian stimulation strategy for women with 17OHD. Although Xu et al. used endogenous progesterone in controlled ovarian stimulation (19), the patient had a high progesterone level of > 30 ng/ml. Our case study reports detailed information on various hormone levels, and conditions suitable for endogenous progesterone stimulation, the data on which is lacking.

In 17OHD cases, elevated progesterone is very common in controlled ovarian stimulation, despite the use of dexamethasone (8, 9, 11, 20). This may be due to the deficiency of 17OH in the developing ovarian follicles. Embryo cryopreservation is the better protocol. In all previous reports of embryo transfer in 17OHD cases, frozen embryo transfer was used for all live births (9, 10, 12, 19). We used a hormone replacement cycle for frozen embryo transfer, and a regimen of lowering endogenous progesterone with dexamethasone plus GnRHa before the transfer. The GnRHa was used to inhibit follicular development and avoid high progesterone levels due to 17OHD in the ovary.

Azziz et al. reported that there were altered enzyme kinetics and overactivation of the renin–angiotensin–aldosterone axis in 21OHD cases (21). Increasing fludrocortisone dosage in patients with increased plasma renin activity and 17a-hydroxyprogesterone concentration resulted in a better control of congenital adrenal hyperplasia hormone disorders (22). In 17OHD, over-secretion of corticosterone, 11-deoxycorticosterone (DOC), 18-hydroxy-DOC, 18-hydroxycorticosterone, and 19-norDOC leads to low-renin hypertension and hypokalaemia (23). Irrespective of whether hypertension is present, glucocorticoids have been used in 17OHD women undergoing IVF, from egg retrieval to delivery, in previous studies (9, 10, 12, 19). In this study, we found that dexamethasone, and not hydrocortisone, effectively reduced the patient’s high progesterone levels. We deduced that hydrocortisone has mineralocorticoid activity, which further inhibits renin–angiotensin, decreases CYP11B2 activity, and leads to increased progesterone accumulation. Another reason may be the longer half-life of dexamethasone and its strong inhibitory effect on the hypothalamus–pituitary–adrenal gland axis.

Dexamethasone can also cause adverse effects during IVF. Ben-Nun et al. reported an IVF pregnancy was terminated due to uncontrolled hypertension at 25 weeks (12). The long-term use of dexamethasone can also cause hypertension and hyperglycemia (24). Foetal malformations can also arise due to dexamethasone use (14). To reduce dexamethasone-related side effects, Xu et al. used dexamethasone before frozen embryo transfer and changed the glucocorticoid to prednisone after embryo transfer. However, the first pregnancy was terminated because the foetus had cleft lip and palate (19). Therefore, we stopped glucocorticoid administration once pregnancy was detected to promote pregnancy and avoid the side effects of administration on foetus. Our patient did not have hypertension or hypokalaemia. We speculate that glucocorticoids are not necessary to maintain pregnancy.

Our case study is not without limitations. Our report is based on a single case and further research with robust study designs are crucial to reinforce our observations.

## Conclusion

In this 17OHD infertile case, use of dexamethasone for IVF reduced glucocorticoid-associated side effects and promoted healthy live births. Glucocorticoids were only used to reduce progesterone levels in the artificial endometrial scheme and were not used during controlled ovary stimulation and post-implantation pregnancy. Our study provides a referable proof for patients with non-classical 17OHD who report basic progesterone level > 2 ng/ml and do not present with hypertension or hypokalaemia. However, owing to the low incidence of 17OHD, more cases are needed for further validation.

## Data availability statement

The raw data supporting the conclusions of this article will be made available by the authors, without undue reservation.

## Ethics statement

The studies involving human participants were reviewed and approved by the ethics committee of Peking university, first hospital. The patients/participants provided their written informed consent to participate in this study.

## Author contributions

X-LY, T-TZ designed the research. X-LY, T-TZ, JS, QX, Y-RK, SW and YX conducted the research. X-LY, T-TZ wrote the article and X-LY had primary responsibility for final content. All authors contributed to the article and approved the submitted version.

## Funding

National High Level Hospital Clinical Research Funding (Interdepartmental Clinical Research Project of Peking University First Hospital) 2022CR04.

## Conflict of interest

The authors declare that the research was conducted in the absence of any commercial or financial relationships that could be construed as a potential conflict of interest.

## Publisher’s note

All claims expressed in this article are solely those of the authors and do not necessarily represent those of their affiliated organizations, or those of the publisher, the editors and the reviewers. Any product that may be evaluated in this article, or claim that may be made by its manufacturer, is not guaranteed or endorsed by the publisher.
